# The Time Course of JNK and P38 Activation in Cerebellar Granule Neurons Following Glucose Deprivation and BDNF Treatment

**Published:** 2012

**Authors:** Niki Vakili Zahir, Mousa Abkhezr, Zahra Khaje Piri, Seyyed Nasser Ostad, Abbass Kebriaezade, Mohammad Hossein Ghahremani

**Affiliations:** Department of Pharmacology-Toxicology, Faculty of Pharmacy, Tehran University of Medical Sciences, Tehran, Iran.

**Keywords:** MAPKs, CGN, Brain-derived neurotrophic factor, Signaling

## Abstract

Low glucose condition induces neuronal cell-death via intracellular mechanisms including mitogen-activated protein kinases (MAPK) signaling pathways. It has been shown that low glucose medium decreases neuronal survival in cerebellar granule neurons (CGNs). In this study, we have examined the activation of JNK, p38kinase and ERK1/2 pathways in low glucose medium in CGNs.

The CGNs were prepared from new-born (P-2 and P-5) rats and cultured in Dulbecco′s Modified Eagle′s Medium high (DMEM-HIGH) glucose supplemented with Fetal Bovine Serum (FBS) 10% for 7 days. The glucose deprivation was induced through replacing the culture medium with the low glucose (5 mM) medium. The MAPK pathways activation was evaluated through phospho specific antibodies using western blot. The viability of cells was measuring using MTT assay.

The results indicated that low glucose reduces the cell survival and brain-derived neurotrophic factor (BDNF) elevates the cell viability in CGNs. The basal c-Jun N-terminal kinase (JNK) activity was high in CGNs and glucose deprivation for 24 h had increased phospho-JNK level to 2-fold compared to basal. BDNF treatment reduced the basal JNK activity within 30 min but had no effect in longer incubations. BDNF also blocked the low glucose-induced JNK activation. In addition, CGNs exhibited high p38 phosphorylation in low glucose medium in 48 h.

These results demonstrated that in sustained low glucose conditions, CGNs had high activity of stress-activated MAPK which could induce cellular damage. Moreover, BDNF can prevent JNK and p38 activation in stress conditions and increase cell viability. Our results suggest that in sustained stress conditions, inhibition of JNK and/or p38 pathways might protect neurons from damage in low glucose conditions.

## Introduction

The molecular mechanisms responsible for intracellular signal transduction of extracellular stimuli provide knowledge in understanding the biological processes involved in disease (1). Hypoglycemic condition has been shown to induce stress as well as cell-death in neurons. However, the mechanisms involved in this model of neuronal death are not fully explained. During the development, brain-derived neurotrophic factor (BDNF) is required for the normal development and maturation of cerebellar granule neurons as well as the survival of certain neuronal population in central and peripheral nervous system (2-4). The mitogen-activated protein kinases (MAPK) pathways have been identified as the key regulators of the cell growth and proliferation, differentiation, and cell-death (5, 6). The c-Jun N-terminal kinases (JNKs, JNK1, 2, 3) and p38 MAP kinases (p38, p38 *α*, *β*, *γ *and *δ*) are stress-activated protein kinases (1, 7), while the extracellular signal-regulated kinases (ERKs, ERK1/2) activate survival responses (1, 6). Activated JNK and p38 can be translocated to the nucleus and can phosphorylate transcription factors such as c-Jun, ATF-2 and Elk-1 (5, 8, 9). It has been shown that the activations of JNK and p38 are involved in various stress-induced neuronal death in CGNs including glutamate-induced neuronal death (10), low potassium-induced neuronal damage (11) and hypoxia-induced cell-death (12, 13). Interestingly, in Alzheimer›s disease, compared to age-matched normal tissue, p38 kinase levels were high in brain tissue (14). Furthermore, it has been shown that BDNF can protect CGNs from stress-induced cell damage (15-18). Considering the fact that one of the major component of stroke related to ischemia is hypoglycemic brain damage (18, 19), the signaling mechanism involved in glucose deprivation-induced death in neurons can identify therapeutic targets to prevent brain damage. In this study we have evaluated the time-course of the activation of JNK, p38 and ERK pathways following glucose deprivation in CGNs and also tested the protective role of BDNF in low glucose conditions.

## Experimental

Cell culture reagents (DMEM, FBS, penicillin-streptomycin, and trypsin) were purchased from Life Technologies Gibco (Technologies Gibco, UK). All cell culture dishes were from SPL (SPL, Korea). Cytosine arabinoside (AraC) and poly-D-lysine were from Sigma-Aldrich (Sigma-Aldrich, USA). Deoxyribonuclease I (DNaseI), BDNF, DTT, Western blot detection kit and Poly vinyl difluoride (PVDF) were obtained from Roche Applied Science (Roche Applied Science, Germany). Phospho-JNK, Phospho-ERK, Phospho-p38, ERK1/2, p38, and JNK antibodies were from Cell Signaling (Cell Signaling, USA) and β-actin antibody was purchased from Santa Cruz (Santa Cruz, USA). Biomax film was obtained from Kodak (Kodak, UK). All the other chemicals were from Merck (Merck, Germany).

*Cerebellar granule cultures*

Cultures enriched in cerebellar granule cells were prepared based on the standard trypsin disaggregation protocol (10, 20). Briefly, cerebellars from 2 , 5 and 7 day-old (P2,P5 and P7) rat pops were isolated, chopped into 1 mm pieces and incubated in a Ca^+2^-Mg^+2^-free PBS containing glucose 1 mg/10 mL, trypsin (0.25%) and DNaseI (100 U/mL) for 10 min in room temperature (RT). The tissues were triturated with flame-polished pipettes and the cell suspensions were filtered through a 40 μm nylon mesh. The cell suspension was then centrifuged at 400 g for 5 min. Neurons were resuspended in Dulbecco›s modified Eagle›s medium supplemented with 10% heat-inactivated fetal bovine serum, 20 mM KCL, 30 mM glucose, and penicillin (20 U/mL)-streptomycin (20 μg/mL) at a density of 1 × 10^6 ^cells/mL and plated onto poly-D-lysine-coated 12-well dishes. Cultures were kept at 37°C in humidified chamber with 5% CO2 up to 7 days *in-vitro *(DIV). Growth of non-neuronal cells was minimized with cytosine arabinoside (AraC, 5 μM) added to the medium 48 h after the plating.

*Glucose deprivation and drug treatment*

All experiments, unless otherwise stated, were carried out with cerebellar granule neurons at 7 DIV. To induce neuronal death, the high glucose medium (HIGH-DMEM) (4.5 g/L, 25 mM) was replaced through the low glucose medium (Low-DMEM) (1 g/L, 5.5 mM). The cell viability of cultured CGNs was assessed in the presence of AraC (10 μM, 24 and 48 h after the seeding), low glucose medium or BDNF (100 ng/mL) via MTT assay.

*Cell viability*

Neuronal viability was quantified with the measurement of reducing 3-(4, 5-dimethylthiazol-2-yl) 2, 5-diphenyl-tetrazolium bromide (MTT) to purple formazan crystals (21). After the treatments, MTT (5 μg/mL) was added to each well and incubated for 4 h at 37°C. Following incubation, the formazan crystals were dissolved in DMSO and the absorbance was determined at 570 nm with references at 690 nm using Anthos 2020 microplate reader (Anthos, Austria). Cell viability was estimated through MTT assay at 24 or 48 h after the AraC treatment or low-glucose condition. Cell viability was also assessed 4 h after the BDNF (100 ng/mL) treatment.

*Western blotting*

Cells were washed with ice-cold PBS, lysed SDS lysis buffer (Tris-HCl pH = 6.8 6.25%, SDS 10%, Glycerol 10%, DTT 5%, Bromophenol Blue 0.25%) and collected through gentle scraping. Samples containing about 20 μg protein lysate were heated at 95°C for 5 min in SDS sample buffer and loaded on a 10% SDS-polyacrylamide gel. After the electrophoresis, proteins were transferred to PVDF membranes immersed in transfer buffer (25 mM Tris-HCL, 192 mM glycine,15% methanol) using a semi-dry transfer system. The membranes were exposed to blocking buffer (Casein1%, Tween-20 0.1%, TBS pH = 8.0) overnight at 4°C .The membranes were incubated with anti-phospho-JNK(1:1000), anti-JNK1/2 (1:1000), anti-phospho-p38(1:1000), anti-p38 (1:1000), anti-phospho-ERK1/2(1:1000), anti-ERK1/2 (1:1000) and anti-*β*-actin (1:10000), overnight at 4°C. After the incubation, membranes were washed three times with blocking solution and incubated with horse-radish peroxidase-conjugated secondary antibody (1:10000) for 1 h at RT. The membranes were washed three times with 0.1% Tween20 in TBS (TBST). The intensity of the bands was analyzed with the chemiluminescence system. The blots were stripped and reporbed through *β*-actin as internal control. The bands were quantified with densitometry analysis using Scion Image (Scion Image, USA). The signals obtained for each protein were normalized to *β*-actin and presented as the mean ± SE of three independent experiments.

*Statistical analysis*

The results of at least three independent experiments were presented as mean ± SE. Data was compared using one-way ANOVA followed by Tukey-Kramer multiple comparisons (Prism 4.0; Graph Pad, USA) and p < 0.05 was statistically considered significant.

## Results and Discussion

*The effect of rats’ age and cultures on the morphology of cerebellar granule cells*

**Figure 1:**
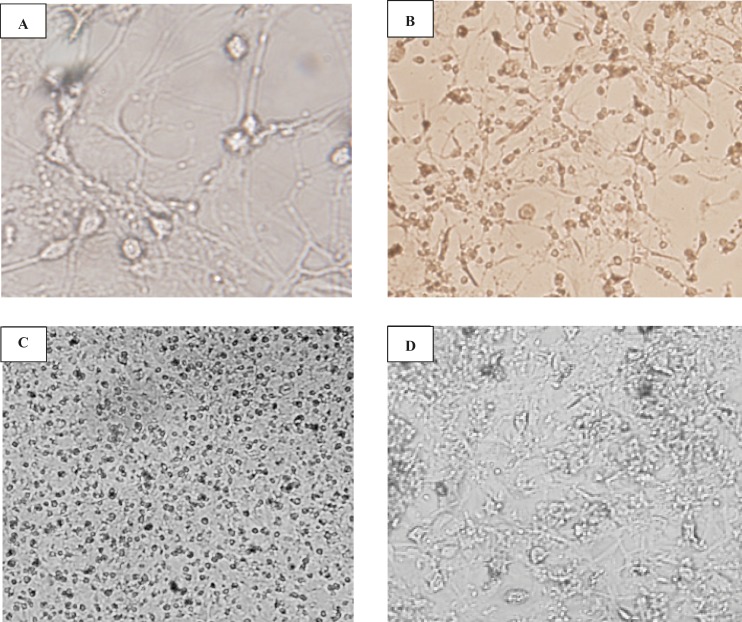
Morphological studies of cerebellar granule cells culture The effect of the age of cultures and rats on the morphological studies on CGN-cultured

When CGNs were cultured in high-glucose (4.5 g/L glucose) and high-potassium (25 mM KCL; K25), neurons were differentiated and survived for more than 2 weeks. Figure 1 shows the typical fields of cerebellar granular cells isolated from P-2, P-5 and P-7 rat pops at 2, 7 and 14 DIV, respectively. As shown, cells have neuronal morphology including large cell bodies and neuritis. These cells showed neuronal phenotype and had tendency to interconnect and made a fine net of axon fibers (Figure 1A, B). As mentioned in methods, in order to minimize the contamination with non-neuronal cells, AraC was added to the media. However, in cells obtained from younger pops, the cell viability was reduced through this adding. We found that the cells at 14 DIV from P-2 cerebellar which was not exposed to AraC, survived for more than two weeks (data is not shown). The same network was observed at 7 DIV of P-5 cerebellar cell culture (Figure 1 C, D), indicating the difference in differentiation stage of the cerebellar granular cells.

Cells were grown in a cultural medium containing DMEM HIGH-glucose and 25 mM (K25). Phase-contrast pictures of cerebellar granule cell cultures at 14DIV (A, D 400x) and 5 DIV (B, C 100x) are shown. Cells isolated from 2-day-old (A, B) and 5-day-old (C, D) pops were grown in DMEM + FBS 10% on poly-D-lysine coated plastic dishes. After 48 h, cells isolated from 5-day-old and 5 DIV (C) were treated with AraC (10 μg/mL). Note the progressive aggregation of cells and the neurite outgrowth.

*Viability of cultured CGNs using MTT assay*

Our results indicated that glucose deprivation induced 30% cell-death in the CGNs culture and BDNF treatment increased cell survival by 30%, compared to the control (Table 1; *F*_2, 8 _= 73.71, p < 0.001). When CGNs were treated with AraC (10 μM) 24 h after the seeding, the cell viability was reduced to 20%, while those treated with AraC (10 μM) 48 h after being cultured had 40% survival (Table 1; *F*_2,6 _= 2676, p < 0.0001). 

**Table 1:** The effect of stress conditions on CGN viability

	**Mean ± SD **	**p-value **
Control	98.3 ± 3.3, n=4	--
BDNF	127.9 ± 9.4, n=4	<0.001
Low glucose	71.1 ± 1.0, n=3	<0.01
Control	100.1 ± 1.0, n=3	--
AraC 24h	20.4 ± 1.4, n=3	<0.001
AraC 48h	38.7 ± 1.7, n=3	<0.001

*The glucose deprivation induces JNK activation in CGNs*

**Figure 2:**
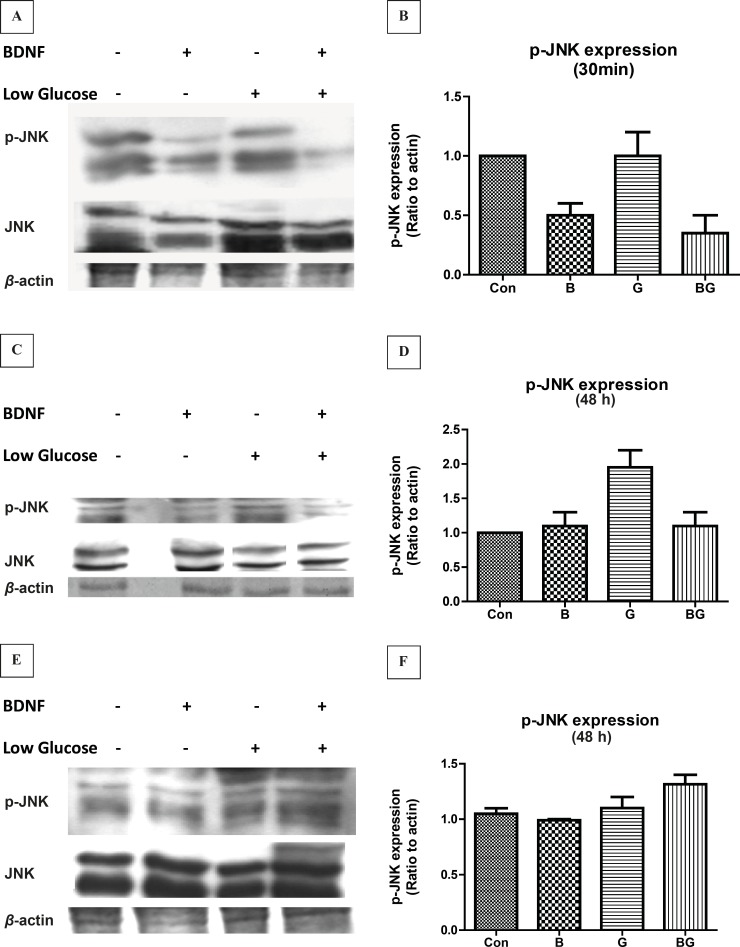
The activation of JNK pathway following glucose deprivation in CGNs. Cells were prepared and cultured in high glucose DMEM + 10% FBS for 7 DIV as described in methods. The CGNs were then treated with BDNF in low glucose medium (low glucose) or normal medium (high glucose) and compared with untreated cells (Control) or cells in low glucose medium. The results of the treatment for 30 min (A, B) , 24 h (C, D) and 48 h (E, F) are shown. Total cell lysate was prepared after the treatment and subjected to SDS-PAGE. The bands for phospho-JNK and total JNK were detected using specific antibodies (A, C, E). Densitometric analyses were performed and protein expressions were calculated as the ratio to *β*-Actin (B, D, F). Data was presented as the mean ± SE of three independent experiments (n = 3, ** p < 0.01, *** p < 0.001).

We investigated the time course of JNK activation in glucose deprivation-induced cell-death in CGNs. Our findings indicated that the level of active JNK was high in cultured CGNs under resting conditions. Glucose deprivation of neurons did not change the phosphorylation of JNK in 30 min. On the other hand, BDNF treatment for 30 min decreased the JNK activation by 50% in basal as well as low glucose conditions (Figure 2 A, B, *F*_3, 8 _= 18.86, p < 0.001). After 24 h of exposure, the low glucose medium increased the phospho-JNK expression in CGNs by 2-fold (Figure 2 C, D; *F*_3, 8 _= 18.10, p < 0.001). BDNF treatment at 24 h partially inhibited the glucose-deprived-induced JNK phosphorylation but had no effect on the basal level. After 48 h treatment, the phosphorylation of p-JNK in CGNs was not significantly different from the basal phosphorylation in any conditions (Figure 2 E, F; *F*_3, 8 _= 3.71, p = 0.058). 

*The low glucose medium induces p38 MAPK activation in CGNs*

**Figure 3:**
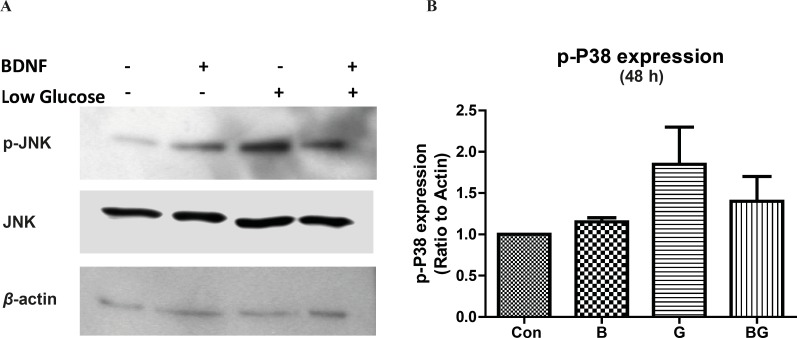
The activation of p38 pathway following glucose deprivation in CGNs Cells were prepared and cultured in high glucose DMEM + 10% FBS for 7 DIV as described in methods. The CGNs were then treated with BDNF in low glucose medium (low glucose) or normal medium (high glucose) and compared with untreated cells (Control) or cells in low glucose medium for 48 h. Total cell lysate was prepared after the treatment and subjected to SDS-PAGE. The bands for phospho-p38 and total p38 were detected using specific antibodies (A). Densitometric analyses were performed and protein expressions were calculated as the ratio to *β*-actin (B). Data was presented as the mean ± SE of three independent experiments (n = 3

We showed that whether the activation of p38 signaling pathway is involved in glucose deprivation-induced cell-death. Our results indicated that glucose deprivation did not change the phosphorylation of p38 kinase in 30 min (data is not shown); however, p38 activation was increased by 2-fold after 24 h exposure to low glucose medium, (data is not shown) and stayed high even after 48 h (Figure 3). Similar to JNK activation, BDNF did not change the basal activation of p38 but lowered the low glucose-induced p38 phosphorylation in 48h (Figure 3 A, B; *F*_3, 8_ = 2.41, p = 0.14). 

*The ERK1/2 pathway is not activated after long term BDNF treatment or in glucose deprivation*

**Figure 4:**
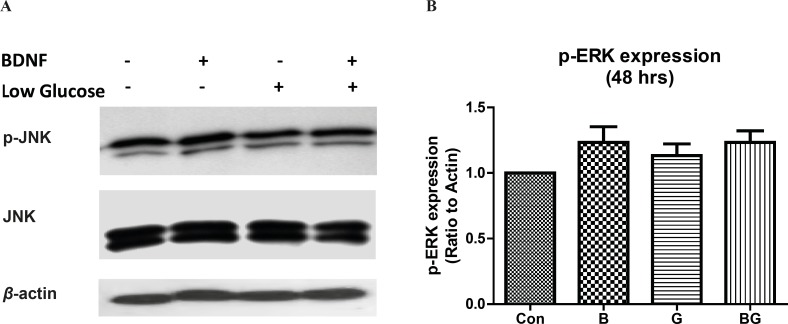
The effect of ERK1/2 activation following glucose deprivation in CGNs. Cells were prepared and cultured in high glucose DMEM + 10% FBS for 7 DIV as described in methods. The CGNs were then treated with BDNF in low glucose medium (low glucose) or normal medium (high glucose) and compared with untreated cells (Control) or cells in low glucose medium for 48 h. Total cell lysate was prepared after the treatment and subjected to SDS-PAGE. The bands for phospho- ERK1/2 and total ERK1/2 were detected using specific antibodies (A). Densitometric analyses were performed and protein expressions were calculated as the ratio to β-Actin (B). Data was presented as the mean ± SE of three independent experiments (n = 3).

In order to evaluate the effect of glucose deprivation on sustained activation of the ERK1/2 pathway in CGNs, we determined the phosphorylation of ERK by western blot. Neither the low glucose conditions nor the BDNF changed the ERK1/2 activation in 48 h (Figure 4; *F*_3, 8 _= 1.63, p = 0.26). Although we observed a decrease in ERK1/2 activity by low glucose at 30 min (data is not shown), this effect was not sustained in longer treatments. 

The function of mitogen-activated protein kinases (MAPKs) has been implied in cellular events ranging from normal proliferation, differentiation and cell-death to diseases including cancer, inflammation and neurodegenerative diseases (23, 24). We have evaluated the time course of JNK activation in CGNs following glucose deprivation and BDNF treatment. Our results indicated that the cerebellar granule neurons express the high basal level of active JNK (phospho-JNK). Changing the culture condition to low glucose increased the JNK activity after 24 h but did not alter JNK phosphorylation in short term (30 min) treatments. Similarly, sustained stress condition induced by low glucose increased the activation of p38 kinase in CGNs (Figure 3). These results point toward a sustained increase in JNK and p38 activity in low glucose medium. On the other hand, BDNF decreased the basal and low glucose JNK activity in 30 min but had no effect on JNK or p38 activity during longer periods. Lack of BDNF activity in long term (24 h) can be related to either short half life of BDNF in culture medium or compensatory cellular mechanisms. In any case, the BDNF inhibition of JNK activation in low glucose medium can be accounted for the neuroprotective effect of BDNF in stress conditions. There are numerous evidences supporting the role of JNK and p38 pathways in regulating cell-death and differentiation in neurons (11, 12, 25, 26). Liu *et al. *have shown that the JNK and p38 activations are involved in hypoxia-induced cell-death in CGNs (12). Yamagishi *et al. *reported the role of p38 kinase in low potassium-induced CGN death (11). Xia *et al. *demonstrated that the activation of JNK in PC12 cells following NGF withdrawal induced neuronal apoptosis (25). In cerebellar granule neurons, deprivation of cells from survival factors, serum or induction of stress in low potassium results in increased c-Jun mRNA level (27) followed by neuronal death via apoptosis. Besides, it has been reported that the inhibition of JNK activation is protective against the low potassium-induced neuronal death in CGNs (28). Interestingly, the ERK1/2 activity, as protective pathway, was low in long-term stress condition. Therefore, our results suggest that the inhibition of JNK or p38 activation can benefit neuronal viability in glucose-deprived condition. 

The high level of phospho-JNK in control cells may be the result of a normal development and/or stress conditions, since most of the cells in this culture are in the process of differentiation. This finding is supported by the fact that CGN cultures from young animals (P2-P5) are sensitive to AraC treatment, as the neurons were not fully differentiated, and BDNF treatment increased the proliferation in these cultures. Moreover, the addition of AraC 48 h after the seeding showed less cell-death, suggesting more resistance to AraC effect due to the neuronal differentiation. It is known that the activity of JNK pathway is increased during the development and differentiation (5, 6). Thus, this high activity can be related to the differentiation process of the cultured cells. 

Taken together, our results point toward a sustained increase in JNK and p38 activation which lasts for 48 h, suggesting the lack of compensatory mechanisms in neuron to adapt with the stress conditions. Thus, in short term, BDNF and block of JNK and/or p38 pathways can be beneficial to stress-induced neuronal death. However, in sustained stress conditions, strategies to inhibit the JNK and p38 kinases can ameliorate the death conditions induced by low glucose conditions.
